# Identification of stabilizing point mutations through mutagenesis of destabilized protein libraries

**DOI:** 10.1016/j.jbc.2022.101785

**Published:** 2022-03-03

**Authors:** Shahbaz Ahmed, Kavyashree Manjunath, Gopinath Chattopadhyay, Raghavan Varadarajan

**Affiliations:** 1 Molecular Biophysics Unit, Indian Institute of Science, Bangalore, India; 2 Centre for Chemical Biology and Therapeutics, Institute of Stem Cell Science and Regenerative Medicine, Bangalore, India

**Keywords:** protein stability, protein structure, protein folding, High-throughput screening (HTS), protein evolution, hyperstable mutants, second-site suppressor mutagenesis, protein engineering, DMS, deep mutational scanning, FACS, fluorescence-activated cell sorting, MFI, mean fluorescent intensity, MID, multiplex identifier, PIM, parent inactivating mutation, RBD, receptor-binding domain, SARS-CoV-2, severe acute respiratory syndrome coronavirus 2, SPR, surface plasmon resonance, SSSM, single-site saturation suppressor mutagenesis, WT, wild-type, YSD, yeast surface display

## Abstract

Although there have been recent transformative advances in the area of protein structure prediction, prediction of point mutations that improve protein stability remains challenging. It is possible to construct and screen large mutant libraries for improved activity or ligand binding. However, reliable screens for mutants that improve protein stability do not yet exist, especially for proteins that are well folded and relatively stable. Here, we demonstrate that incorporation of a single, specific, destabilizing mutation termed parent inactivating mutation into each member of a single-site saturation mutagenesis library, followed by screening for suppressors, allows for robust and accurate identification of stabilizing mutations. We carried out fluorescence-activated cell sorting of such a yeast surface display, saturation suppressor library of the bacterial toxin CcdB, followed by deep sequencing of sorted populations. We found that multiple stabilizing mutations could be identified after a single round of sorting. In addition, multiple libraries with different parent inactivating mutations could be pooled and simultaneously screened to further enhance the accuracy of identification of stabilizing mutations. Finally, we show that individual stabilizing mutations could be combined to result in a multi-mutant that demonstrated an increase in thermal melting temperature of about 20 °C, and that displayed enhanced tolerance to high temperature exposure. We conclude that as this method is robust and employs small library sizes, it can be readily extended to other display and screening formats to rapidly isolate stabilized protein mutants.

Directed evolution has drastically reduced the time required to engineer desired functions into proteins (1, 2, 3, 4). Enzymes and other proteins with altered function or binding specificity have been evolved using yeast surface display (YSD), phage display (5, 6) or other *in vivo* functional screens (7, 8, 9). Phage display utilizes its surface proteins pIII and pVIII, which are fused to the protein of interest (10). Phage display can be used to generate libraries of very high diversity (11) which can be screened for binding to a target ligand. Agglutinin-based Aga2p is a widely used system to display proteins on the yeast cell surface (5). Aga2p is a small protein, covalently linked *via* disulfide linkages to the yeast cell surface protein Aga1 (12). Different populations in a yeast library can be enriched using fluorescence-activated cell sorting (FACS). Relative to the phage display library, sizes are lower (∼10^11^ *versus* 10^7^). However, with YSD, eukaryotic post-translation modifications are possible. While screening for mutants with improved binding or enzymatic activity is straight forward, it is nontrivial to screen for mutants with improved stability. Deep mutational scanning (DMS) is an approach that combines screening/selection of a mutant library with next-generation sequencing to identify the degree of enrichment of mutants following a selection or screen, relative to the population present in the original library (13, 14, 15, 16). Some prior studies have suggested that stabilized mutants are expressed at higher levels than wild type (WT); however, several other studies have not observed this (17, 18, 19). In several cases it was observed that for stable proteins, mutants with improved stability are expressed at a similar level to WT (18, 19, 20, 21) and hence expression alone cannot be used to discriminate mutants with higher stability from mutants with a slightly destabilized phenotype. We recently showed that the amount of active protein on the yeast cell surface (detected by the amount of bound ligand) correlates better with *in vitro* thermal stability or *in vivo* solubility than the amount of total protein on the yeast cell surface, for destabilized mutants (22). However, mutants above a certain stability threshold show similar expression and ligand binding to WT irrespective of their stability. Previously, to find stabilized variants of proteins with high intrinsic stability, YSD libraries were subjected to thermal stress, to enrich for more stable variants followed by sorting to identify variants which retained binding to a conformation-specific ligand (23, 24). While this is potentially useful, yeast cells cannot replicate after high temperature exposure, and hence, the method requires repeated rounds of plasmid isolation, PCR amplification, and retransformation in yeast cells after each round of enrichment. Also, if a protein exhibits reversible thermal unfolding, enrichment of stabilized mutants in such cases will be difficult. An alternative approach to isolate stabilizing mutations is to introduce a destabilizing mutation, hereafter referred to as parent inactivating mutation (PIM), and then create mutant libraries in this background to screen for suppressors (25, 26, 27, 28, 29, 30, 31). Often, this methodology requires multiple rounds of enrichment to isolate stable mutants. The reversion of PIM to WT or nondestabilizing mutants during library generation or enrichment enriches for mutants lacking the PIM instead of desired PIM-suppressor pairs. Additionally, in multiround format, this methodology potentially allows isolation of only a few stabilizing mutations and does not distinguish between allele-specific and global suppressors. It is also unclear whether it will always be possible to isolate suppressors for every PIM. In the present study, we have modified this approach by introducing PIMs in the background of a DMS library of bacterial toxin CcdB, sorted different populations, subjected each population to deep sequencing of the CcdB gene, and reconstructed the mean fluorescent intensity (MFI) of each mutant as described (22) (see also Experimental procedures section). We use both the reconstructed binding MFI (MFI_seq_ (bind)) and expression MFI (MFI_seq_ (expr)) as criteria to differentiate between stabilized, WT-like, and destabilized mutants. This single-site saturation suppressor mutagenesis (SSSM) methodology (Fig. 1) was described previously (29). Using this methodology, two different types of suppressors can be identified. Proximal suppressors reverse the destabilizing effect of the PIM by locally compensating packing defects caused by the PIM. Proximal suppressors are allele specific and do not show stabilizing effects as a single mutant in the absence of the PIM (29, 32). Distal suppressors are located far from the PIM and often act as global suppressors, reversing the effects of multiple individual PIMs. A distal suppressor also typically stabilizes the protein relative to WT, in the absence of the PIM. Using this methodology, we could readily identify putative stabilized mutants with a minimal number of false positives.

**Figure 1:**
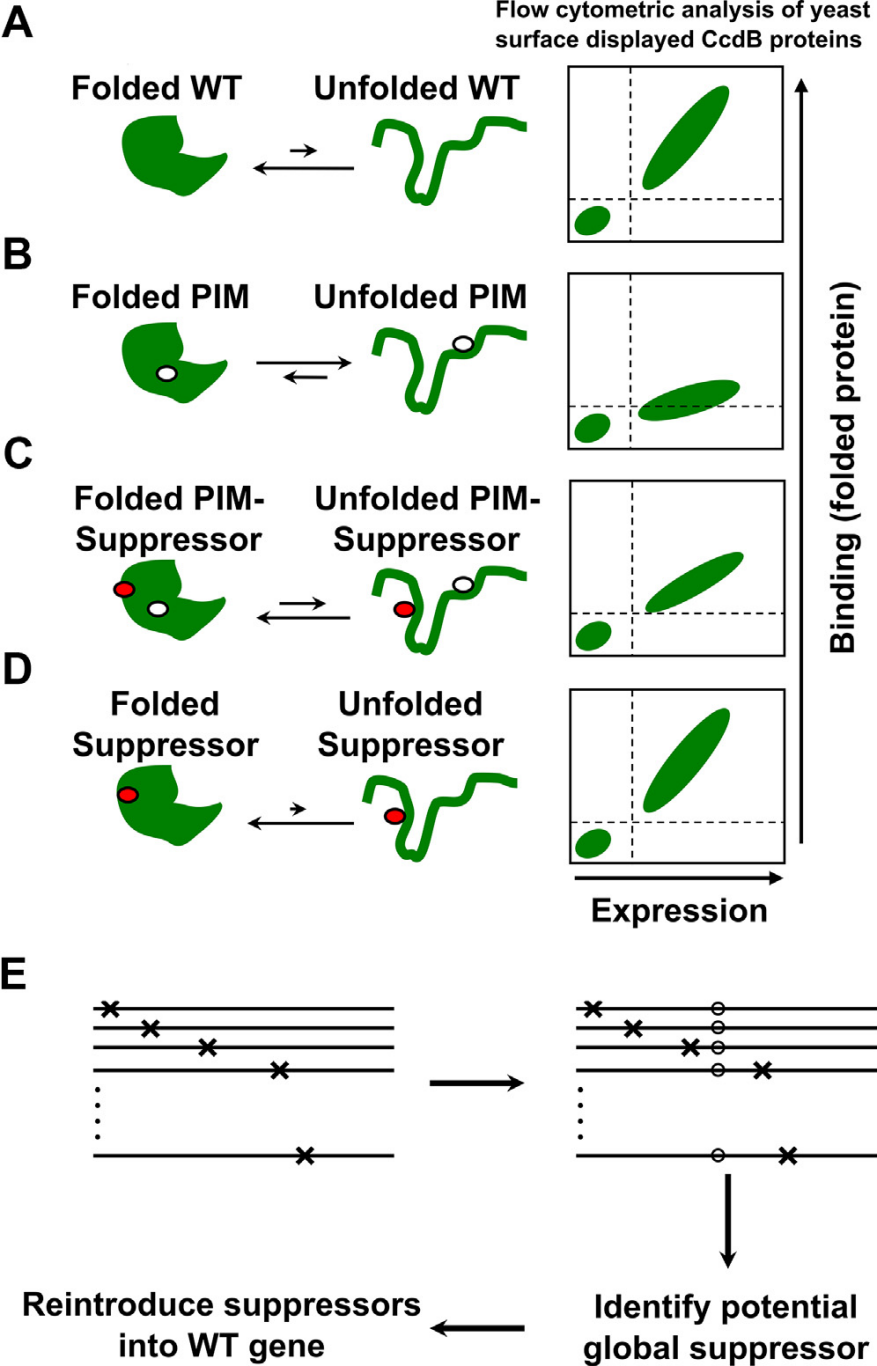
**Schematic representation ofsingle-sitesaturationsuppressor mutagenesis (SSSM) methodology.** Proteins exist in an equilibrium between folded and unfolded states. *A*, WT proteins generally have the equilibrium shifted toward the folded state. Such proteins when expressed on the yeast cell surface show good expression and binding to their cognate ligand. *B*, Introduction of a parent inactivating mutation (PIM) shifts the equilibrium toward the unfolded state and decreases the amount of properly folded protein on the yeast cell surface, leading to decreased ligand binding. *C*, Second-site suppressor mutation, distal from and present in the background of the PIM, will reduce the amount of unfolded protein present at equilibrium. Such double mutants have higher expression and binding on the yeast cell surface compared to the PIM alone. *D*, Such global/distal suppressor mutations can stabilize multiple PIMs and also stabilize the WT protein, although the expression and binding of suppressor alone on the yeast cell surface is similar to the WT protein. *E*, Saturation suppressor libraries are created by introducing a PIM (o) into the background of a deep mutational scanning library, selecting for suppressors, and reintroducing identified suppressors in the background of the WT gene.

## Results

### Selection of parent inactivating mutation and sorting of single-site saturation suppressor mutagenesis library of CcdB

**Figure 2:**
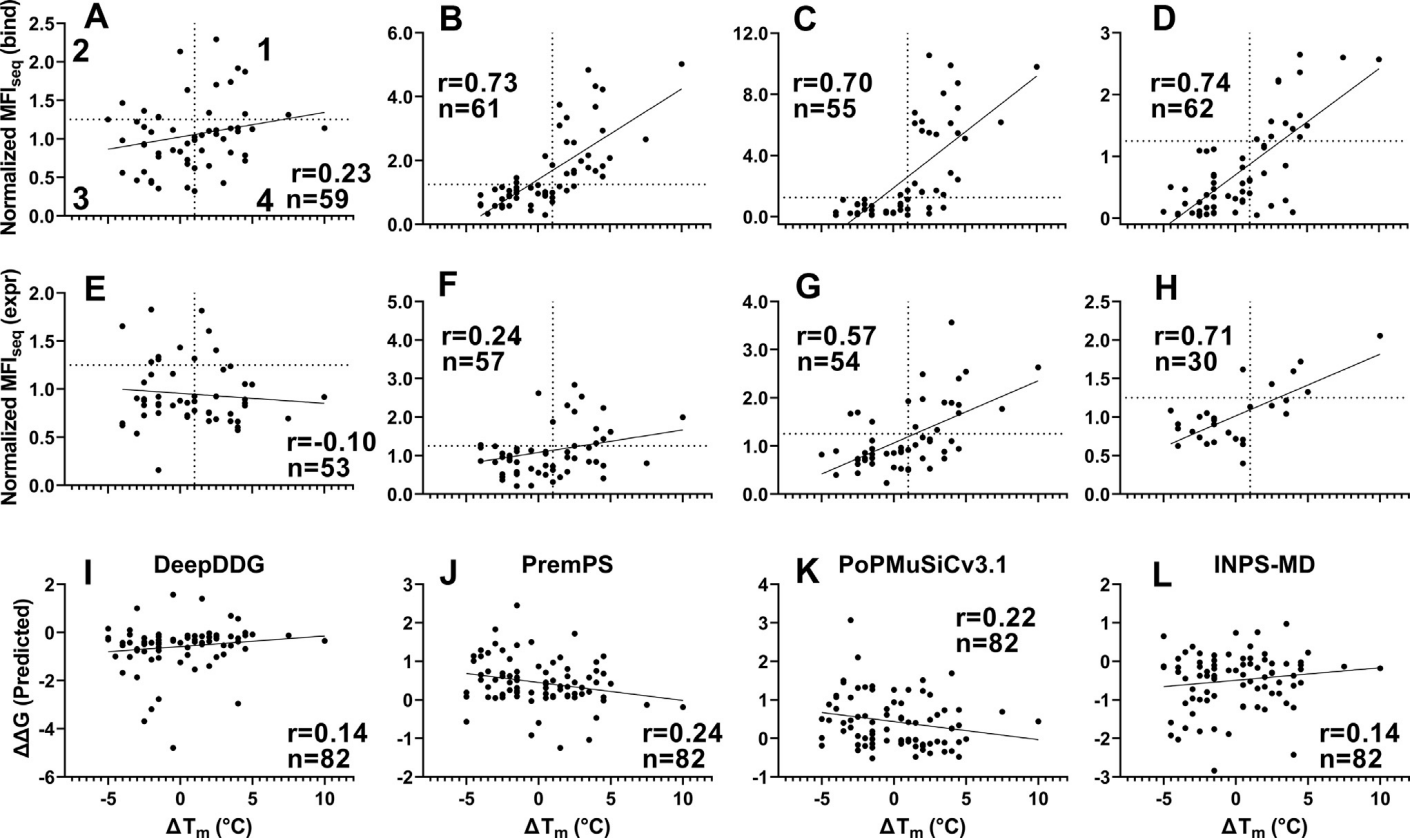
**Correlation of CcdB mutant stability (Δ*T***_**m**_**of single mutant) with normalized MFI**_**seq**_**(bind) or MFI**_**seq**_**(expr) of (PIM, mutant) pair in PIM libraries.** MFI_seq_ values of double mutants were normalized with the MFI_seq_ values of their respective PIM. First, second, third, and fourth quadrants numbered in (*A*) represent true positive, false positive, true negative, and false negative points, respectively. Normalized MFI_seq_ (bind) correlation with thermal stability for V18D (*A*), V18G (*B*), V20G (*C*), and L36A (*D*) libraries. Normalized MFI_seq_ (expr) correlation with thermal stability for V18D (*E*), V18G (F), V20G (*G*), and L36A (H) libraries. Normalized MFI_seq_ (bind) correlates better than normalized MFI_seq_ (expr) with thermal stability. For V18D, while overall correlation is poor, those mutants with the highest MFI_seq_ (bind) are stabilized. Thermal stability predictions by *in silico* methods (*I*) DeepDDG, (*J*) PremPS, (*K*) PoPMuSiCv3.1, and (*L*) INPS-MD. For *in silico* stability measurements, mutants which were present in any library were used. Predicted ΔΔG >0 is stabilizing in the case of DeepDDG and INPS-MD. Predicted ΔΔG <0 is stabilizing in the case of PremPS and PoPMuSiC. MFI, mean fluorescent intensity; PIM, parent inactivating mutation.

CcdB is a bacterial toxin that causes bacterial cell death by binding and poisoning DNA gyrase (33). When expressed on the surface of yeast, properly folded CcdB can be detected by binding to FLAG-tagged GyrA14 followed by incubations of cells with anti-FLAG primary and fluorescently labeled secondary antibodies (29). We selected four PIMs based on their *in vitro* thermal stability and *in vivo* activity in *Escherichia coli* (14, 29). PIM V18D is completely inactive and highly aggregation prone due to a charged mutation in the core of the protein. V18G and V20G are partially inactive *in vitro* due to the formation of a cavity in the core of the protein, with V20G showing higher activity *in vivo* than V18G (14). L36A is the most active among the four PIMs; the mutant protein is partially aggregated (29). SSSM libraries were constructed by introducing each PIM individually in the background of the DMS library (29). These PIMs and their corresponding SSSM libraries showed variable expression and binding depending on the PIM present in the single mutant library (Fig. S1). In each library, binding and expression experiments had slightly different numbers of mutants for which MFI_seq_ was calculated (Table S1). Different populations of SSSM libraries were sorted based on the expression and binding histograms of the libraries (Fig. S2). Populations were subjected to deep sequencing, and the mean fluorescence intensities of binding (MFI_seq_ (bind)) and expression (MFI_seq_ (expr)) were estimated as described (22). Values of normalized MFI_seq_ (bind) and normalized MFI_seq_ (expr) were used to predict stabilized mutants. We only considered mutants that were present in more than one library. Putative stabilized mutants were identified as those that have a normalized MFI_seq_ value for either binding or expression >1.25. WT-like mutants were those that had normalized MFI_seq_ values of 0.9 to 1.25, and destabilized mutants were those that had normalized MFI_seq_ values of <0.9. Within each predicted class, mutants were randomly selected for experimental validation. One hundred twelve individual point mutants in the WT background were expressed and purified, and the *T*_m_ was measured using a thermal shift assay (Table S2). We found a better correlation between the MFI_seq_ (bind) and the stability of stabilized and marginally destabilized (0>Δ*T*_m_>−5) mutants than between MFI_seq_ (expr) and the stability of the mutants for all the libraries (Fig. 2, *A*–*H*). In the case of V18D library, the overall correlation between MFI_seq_ values of expression or binding with stability of the mutants was poor (Fig. 2, *A* and *E*); nevertheless, the best binders showed significant stabilization. The remaining libraries showed a good correlation of stability with MFI_seq_ (bind) or MFI_seq_ (expr); MFI_seq_ (bind) showed a better correlation than MFI_seq_ (expr) in all the cases.

In a previous report, we compared the accuracy of predictions from several *in silico* tools with our experimental method to estimate the relative stability for several destabilized mutants of CcdB (22). Four *in silico* tools, DeepDDG (34), PremPS (35), PoPMuSiC (36) and INPS-MD (37) which calculated the ΔΔG of mutant (ΔG of unfolding of mutant–ΔG of unfolding of WT) of CcdB mutants showed a correlation of greater than 0.5 with the corresponding *in vitro* measured thermal stability. However, in the case of marginally destabilized and stabilized mutants examined in the present work, the predictions from these programs showed very poor correlation with experimental thermal stability (Fig. 2,*I*–*L*).

In related work, we have stabilized the receptor-binding domain (RBD) of the spike S protein of severe acute respiratory syndrome coronavirus 2 (SARS-CoV-2) using saturation suppressor mutagenesis (38). We identified several stabilizing mutations and could stabilize the protein by 8 °C. We used the PROSS server (see Discussion for details) to predict stabilizing mutations in the RBDPROSS provided only two designs. Design 1 contained the S375D mutation, and design 2 contained S375D and V362I; from analysis of the corresponding YSD data, both of these variants are predicted to have stabilities similar to or lower than that of WT (39). The poor performance of PROSS in this case is probably because of the lack of sufficiently diverse sequence data to identify stabilizing mutations. In the case of CcdB, PROSS predicted several mutations to be stabilizing; however, some were likely to be destabilizing according to our YSD data. Several predictions could not be validated as either they were part of the active site or we did not have corresponding YSD-binding data for the mutant. The PROSS results for CcdB are now summarized in Table S3.

**Figure 3:**
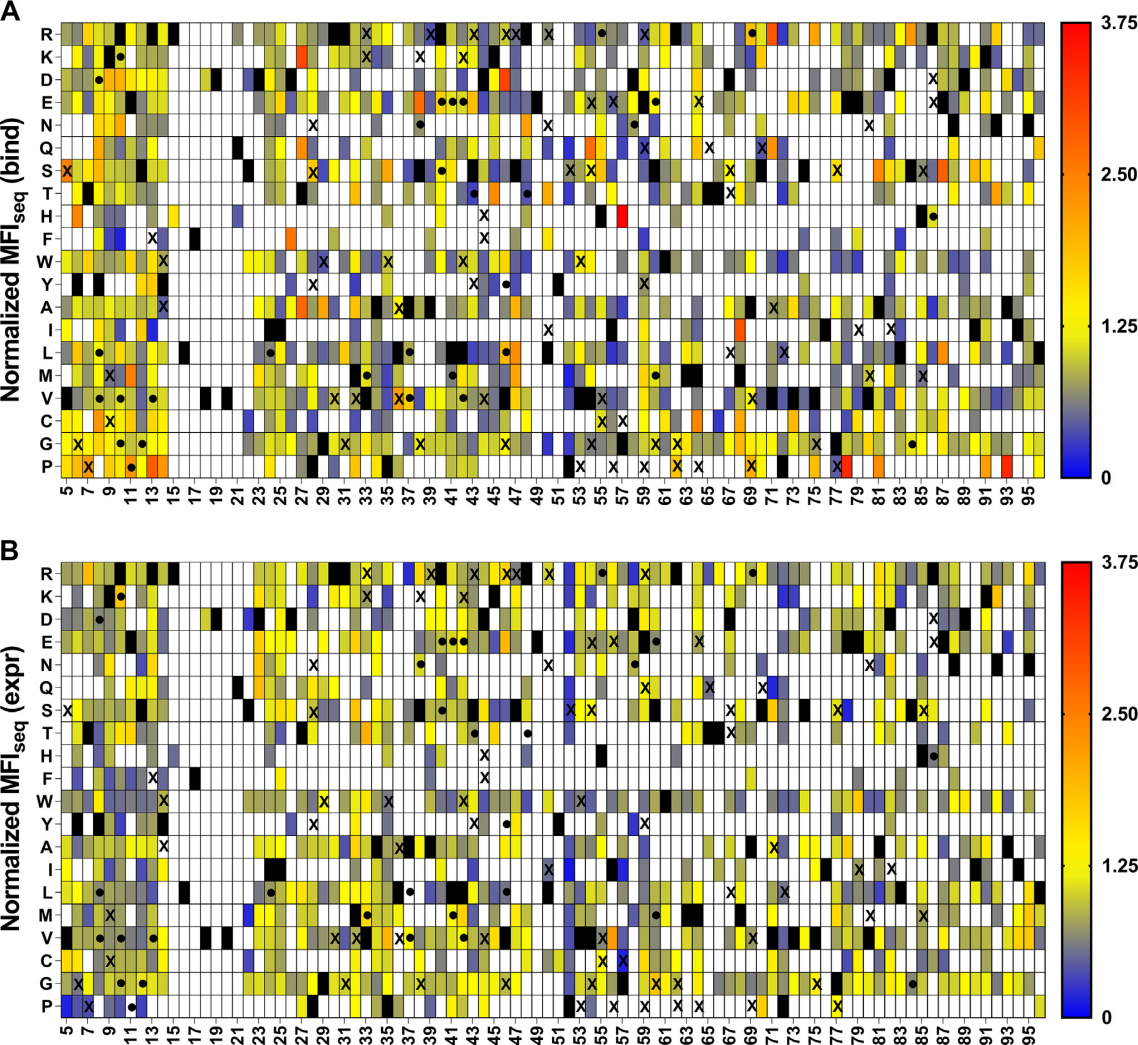
**Heat maps of normalized MFI**_**seq**_**(bind) and MFI**_**seq**_**(expr) for V18D library.** Normalized (with respect to V18D) values of MFI_seq_ (bind) (*A*) and MFI_seq_ (expr) (*B*) were categorized in different ranges. Mutants with normalized MFI_seq_ (bind) or MFI_seq_ (expr) >1.25 were categorized as putative stabilized mutants. *Black rectangle* represents WT residue, and mutants with no data available are indicated with a *white rectangle*. Several single mutants were characterized *in vitro* to estimate their stability, stabilized mutants having Δ*T*_m_>1 are indicated with a “●,” and destabilized mutants Δ*T*_m_<0 are indicated with an “X”. Mutants with normalized MFI_seq_ value ≥3.75 are colored in *red*. MFI, mean fluorescent intensity.

In case of the V18D library, we observed that most mutations have low values of normalized MFI_seq_ (bind) as well as MFI_seq_ (expr). This might be because highly destabilizing PIM cannot be rescued by a single suppressor mutation. We observed a low sensitivity and specificity but reasonable accuracy of prediction for stabilized mutants when predictions were made based on either MFI_seq_ (bind) or MFI_seq_ (expr) (Fig. 3 and Table 1) for this library. Since the results were not as promising as the other libraries, the V18D library was not included in subsequent analyses. A similar analysis was performed for the other three libraries, and we found a high sensitivity of prediction for stabilized mutants for these libraries when MFI_seq_ (bind) was used as the criterion (Table 1).

**Table 1:** Identification of stabilized mutant solely from normalized MFI_seq_ (bind) or MFI_seq_ (expr) data

Parameter	V18D library		V18G library		V20G library		L36A library	
	Bind	Expr	Bind	Expr	Bind	Expr	Bind	Expr
Sensitivity^a^	0.27	0.15	0.80	0.44	0.79	0.54	0.63	0.56
Specificity^a^	0.73	0.88	0.89	0.79	0.98	0.78	1.00	0.91
Accuracy^a^	0.57	0.63	0.86	0.67	0.90	0.70	0.89	0.84

Mutants were predicted to be stabilized if the corresponding MFI_seq_ value was greater than 25%. CcdB mutant thermal stability data were taken from previous studies (22, 54) as well as additional mutant stability data measured in this study (Table S2).

$\text{Sensitivity }=\;\frac{TP}{TP+FN}$

$\text{Specificity }=\;\frac{TN}{TN+FP}$

$\text{Accuracy }=\;\frac{TP+TN}{TP+TN+FP+FN}$

a The sensitivity, specificity, and accuracy were calculated using the following formulae, where TP, TN, FP, and FN correspond to true positive (ΔT_m_ (predicted) > 0, ΔT_m_ (observed) > 0), true negative (ΔT_m_ (predicted) < 0, ΔT_m_ (observed)< 0), false positive (ΔT_m_ (predicted) > 0, ΔT_m_ (observed) < 0), and false negative (ΔT_m_ (predicted) < 0, ΔT_m_ (observed) > 0), respectively.

**Figure 4:**
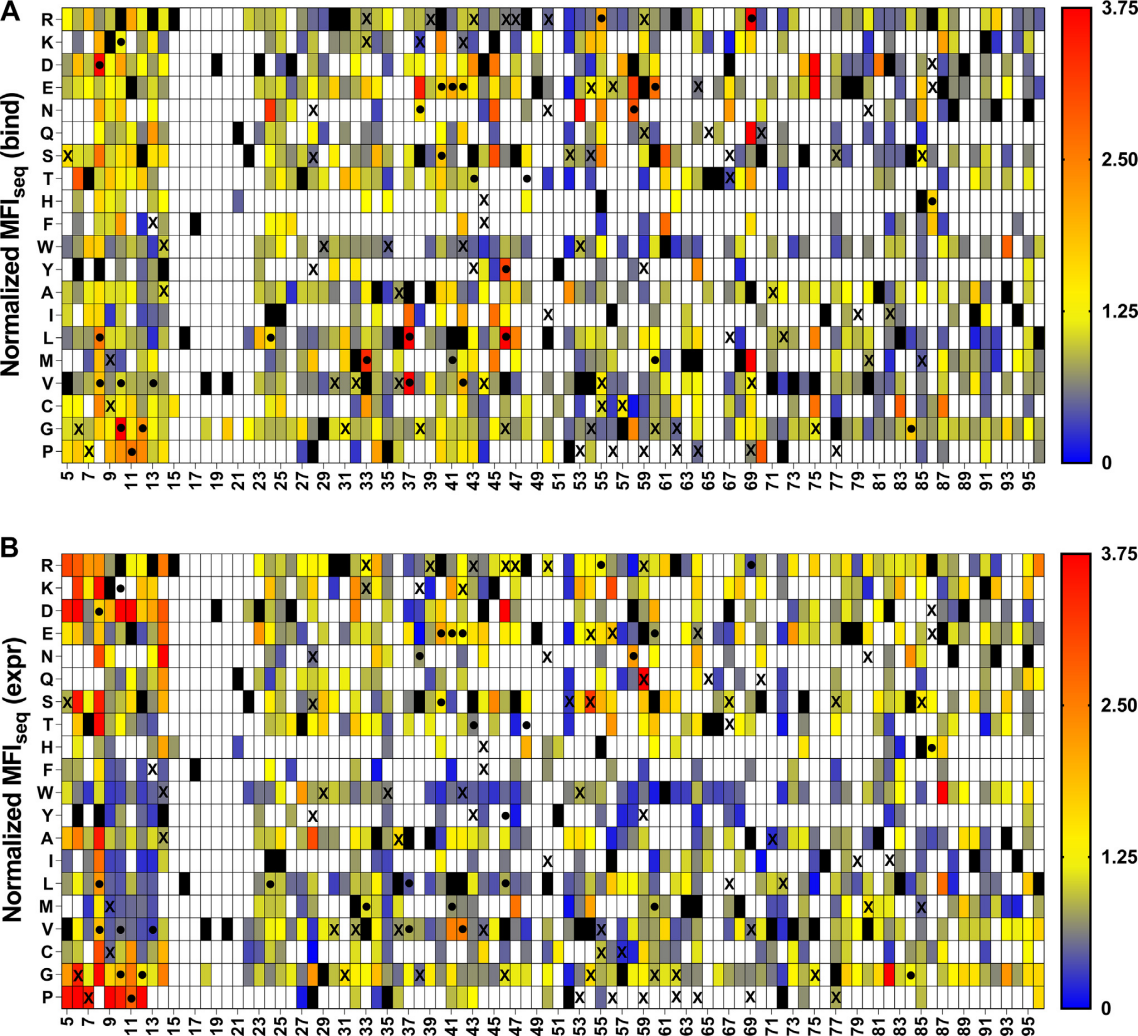
**Heat maps of normalized MFI**_**seq**_**(bind) and MFI**_**seq**_**(expr) for V18G library.** Normalized (with respect to V18G) values of MFI_seq_ (bind) (*A*) and MFI_seq_ (expr) (*B*) were categorized in different ranges. Mutants with normalized MFI_seq_ (bind) or MFI_seq_ (expr) >1.25 were categorized as putative stabilized mutants. *Black rectangle* represents WT residue, and mutants with no data available are indicated with a *white rectangle*. Several single mutants were characterized *in vitro* to estimate their stability, stabilized mutants (Δ*T*_m_>1) are indicated with a “●,” and destabilized mutants with (Δ*T*_m_<0) are indicated with an “X”. Mutants with normalized MFI_seq_ value ≥3.75 are colored in red. MFI, mean fluorescent intensity.

**Figure 5:**
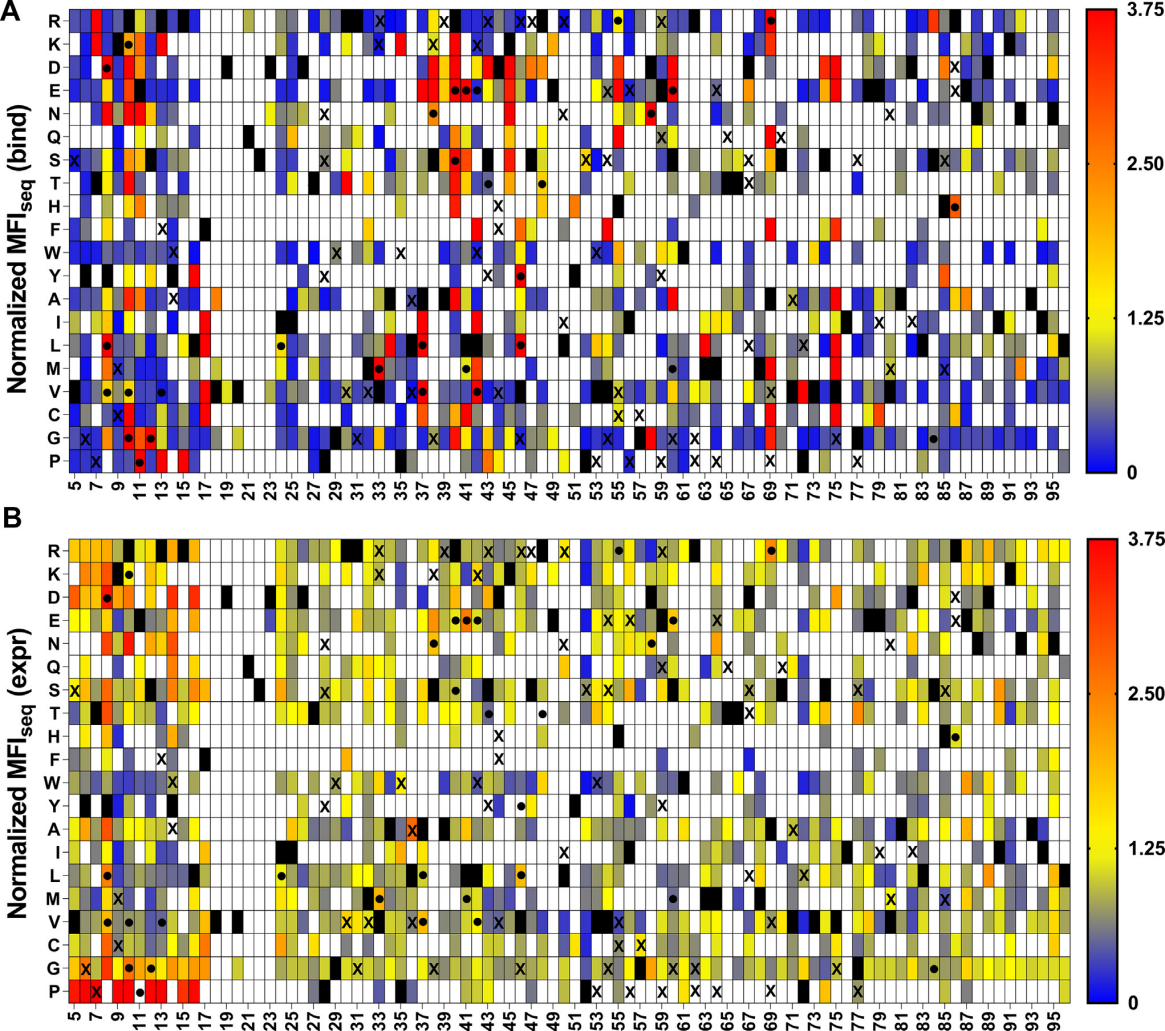
**Heat map of normalized MFI**_**seq**_**(bind) and normalized MFI**_**seq**_**(expr) for V20G library.** Normalized (with respect to V20G) values of MFI_seq_ (bind) (*A*) and MFI_seq_ (expr) (*B*) were colored from *blue* to *red* as increasing MFI_seq_ values. Mutants with normalized MFI_seq_ (bind) or MFI_seq_ (expr) greater than 1.25 were categorized as putative stabilized mutants. *Black rectangle* represents WT residues, and mutants where no data are available are indicated with a *white rectangle*. A subset of mutants was purified, and their *in vitro* thermal stability (*T*_m_) was measured. Stable mutants (Δ*T*_m_>1) are indicated with a “●,” and destabilized mutants (Δ*T*_m_<0) are indicated with an “X”. Mutants with normalized MFI_seq_ value ≥3.75 are colored in *red*. It is clear that MFI_seq_ (bind) is superior to MFI_seq_ (expr) in identification of stabilized mutants. MFI, mean fluorescent intensity.

**Figure 6:**
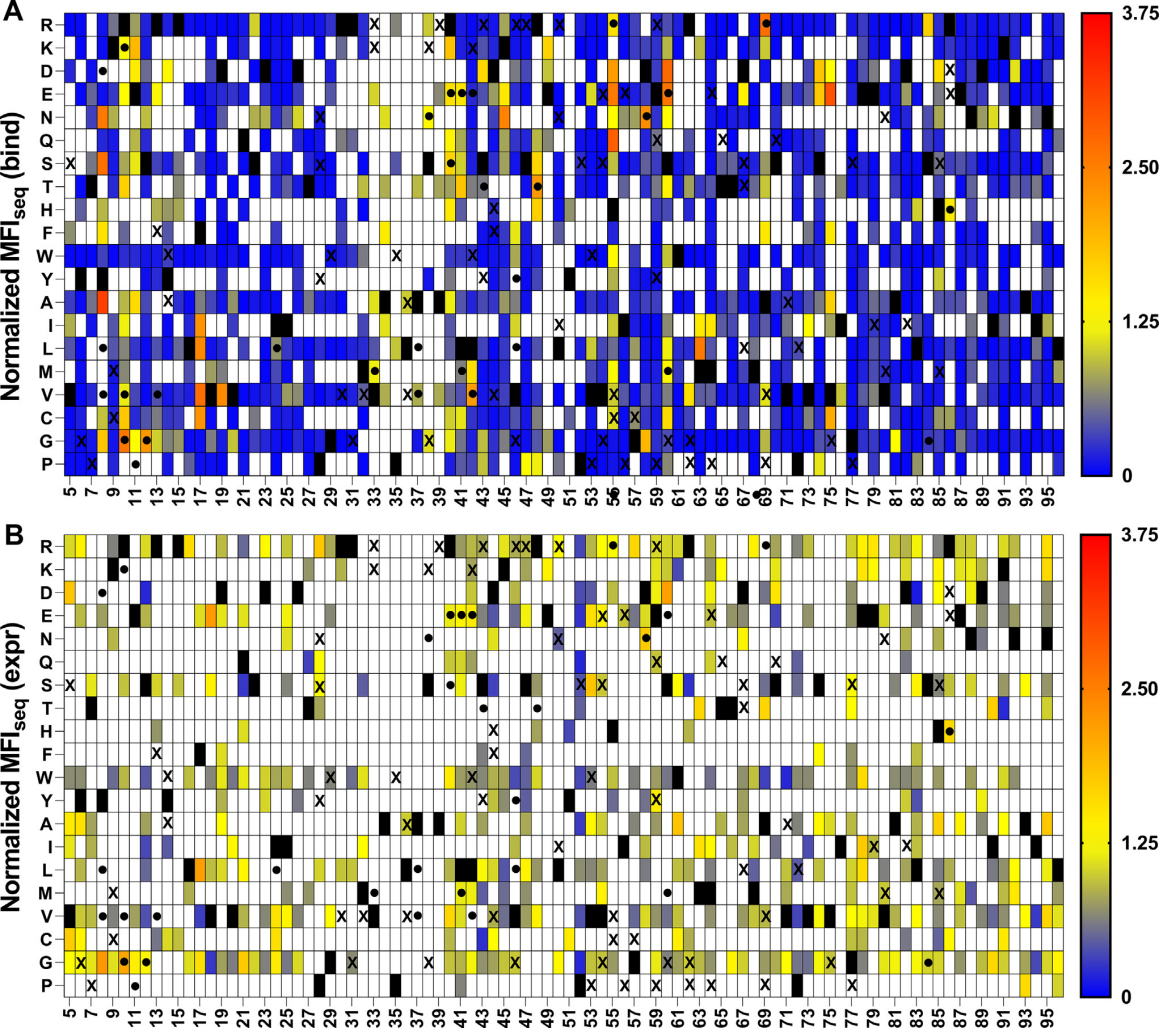
**Heat maps of normalized MFI**_**seq**_**(bind) and MFI**_**seq**_**(expr) for L36A library.** Normalized (with respect to L36A) values of MFI_seq_ (bind) (*A*) and MFI_seq_ (expr) (*B*) were categorized in different ranges. Mutants with normalized MFI_seq_ (bind) or MFI_seq_ (expr) >1.25 were categorized as putative stabilized mutants. *Black rectangle* represents WT residue, and mutants with no data available are indicated with a *white rectangle*. Several single mutants were characterized *in vitro* to estimate their stability, stabilized mutants (Δ*T*_m_>1) are indicated with a “●,” and destabilized mutants with (Δ*T*_m_<0) are indicated with an “X”. Mutants with normalized MFI_seq_ value ≥3.75 are colored in red. MFI, mean fluorescent intensity.

SSSM libraries with V18G or V20G as PIM displayed the highest sensitivity of prediction of stabilizing mutations (Figs. 4 and 5). We found some false positives for the L36A SSSM library (Fig. 6), which had similar expression and binding compared to the DMS library of WT CcdB (Fig. S1). When stabilized mutant predictions were made based on the MFI_seq_ (expr), we observed lower sensitivity compared to the predictions based on MFI_seq_ (bind) (Table 1) for all libraries.

**Figure 7:**
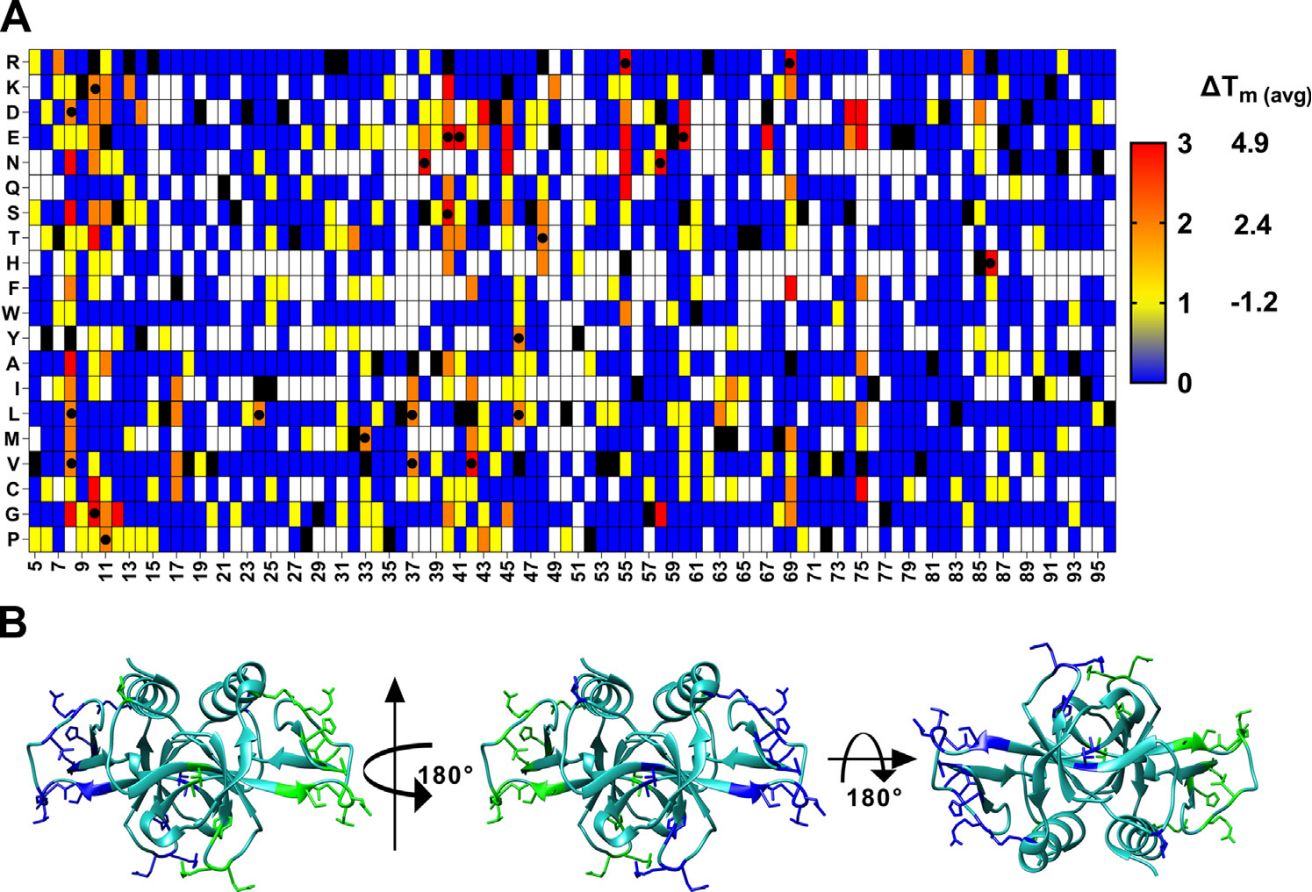
**Putative stabilized mutants found in all the three V18G, V20G, and L36A libraries based on MFI**_**seq**_**(bind).***A*, Heat maps of putative suppressor mutants in each library were given a score of one unit. *Blue* to *red* indicate that the mutant acted as a suppressor in zero, one, two, and three different libraries. *White* indicates the mutants where no data are available. Experimentally confirmed stable mutants (ΔT_m_>1) are indicated with a “●.” *B*, The residue locations of experimentally characterized stabilized mutants are shown in *blue* and *green* color for chain A and chain B of CcdB dimer, respectively (PDB ID: 3VUB). MFI, mean fluorescent intensity.

We hypothesized that the second mutation which alleviates the destabilizing effect of a PIM may act as a global suppressor and can therefore alleviate the destabilizing effects of other PIMs. To confirm this, common suppressors were shortlisted which were present in different PIM libraries (Fig. 7*A*). True global suppressors were identified as those that alleviated the destabilizing effect of at least two PIMs. Using this criterion to identify stabilized mutants, we found a sensitivity of 1 for the prediction. We also found that at several positions, there were multiple mutants with stabilizing phenotypes, which indicates that the WT is not the most preferred in terms of stability; this information can also be used as a criterion to find the stabilizing mutation. Interestingly, none of the suppressors were found at the residues directly involved in GyrA binding. In any case, we have recently shown that such active-site residues can be identified based on the pattern of MFI_seq_ (bind) and MFI_seq_ (expr) in DMS libraries and removed from the set of putative global suppressors (22).

Most of the stabilizing mutations occur in surface-exposed loop regions (Fig. 7*B*), and a proper interpretation is only possible in the context of a high-resolution structure. In another study, where we have carried out detailed mechanistic studies to understand the mechanistic basis for global suppressors in multiple protein systems, we have solved the structures of a few individual stabilizing mutations (S12G, V46L, and S60E) identified in the present study (40). In the case of S12G, we found that two additional water molecules were present in the place of S12 and formed hydrogen bonds with the main chain of residue E11 and R13. In the case of V46L, a new hydrophobic interaction was introduced between side chains of M64 and V46L, as well as an additional hydrogen bond between the R62 side chain and the main chain carbonyl group of residue 46. In the case of WT, a salt bridge is formed between R48 and E49, upon introduction of the S60E mutation, a loop flipping is observed, and R48 now forms a new salt bridge with E60, which restricts the movement of the loop 41 to 50. It is not possible to anticipate these structural changes upon mutation in these mutants through modeling. We estimated the affinity of some of the stabilized and destabilized and multi-mutants of CcdB toward GyrA14 using surface plasmon resonance (SPR) and found that they retain affinities similar to WT CcdB, indicating that mutations at the non–active-site positions do not alter the affinity of CcdB mutants for GyrA14 (Fig. S3).

### Additive effect of stabilizing mutations

**Figure 8:**
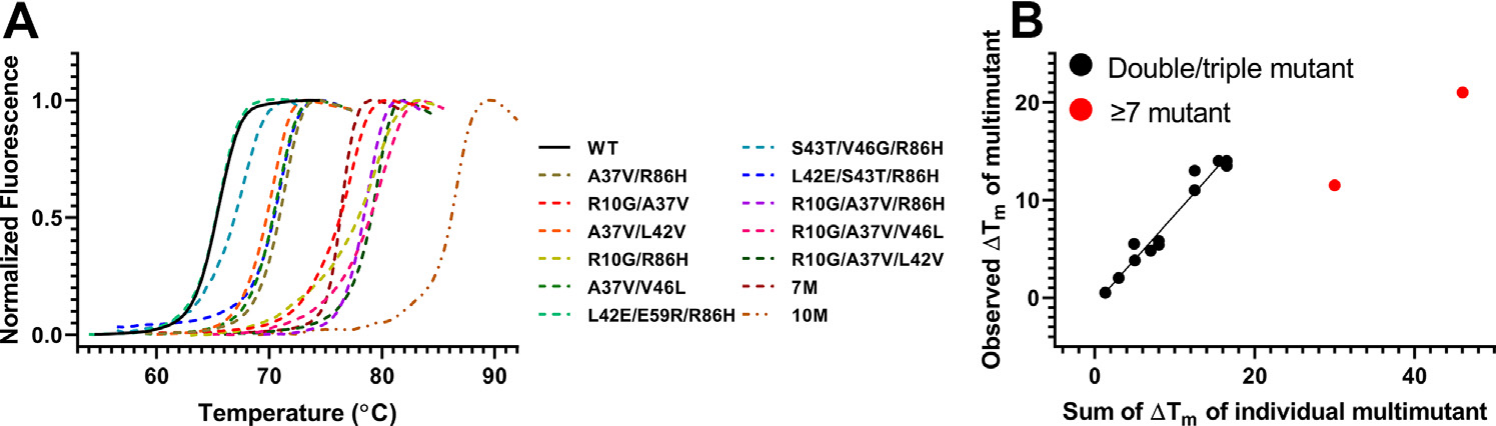
**Thermal shift assay data for select CcdB double, triple, and multi-site mutants.***A*, Data for WT and mutants are shown in *black* and color, respectively. Multi-mutants showed higher thermal stability than individual mutants. Mutations present in 7M are Y8D/R10G/E11P/S12G/A37V/R40S/A69R and 10M are Y8D/R10G/E11P/S12G/A37V/R40S/L42V/V46L/A69R/R86H. *B*, The multi-mutants showed an additive effect when two or three mutations were combined. Multi-mutants that contain seven or more mutations did not show completely additive stabilization.

While single mutations usually do not enhance the stability of proteins to a great extent, individual stabilizing mutations can be combined resulting in multi-mutants with enhanced stability. We therefore constructed multiple double and triple mutants and measured their thermal stabilities using a thermal shift assay. For the generation of double and triple mutants, we used only a single criterion, namely that the centroid–centroid distance between any two of the mutants should be greater than 7 Å. All the double mutants, triple mutants, and multi-mutants showed a higher *T*_m_ than the WT (Fig. 8*A*). We also observed a good correlation (r =0.98) between Δ*T*_m_ of double/triple mutants with the sum of Δ*T*_m_ of individual mutants (Fig. 8*B*). When more than seven mutants were combined, such additive effects were not observed. While combining seven or more stabilizing mutations, we did not consider any centroid–centroid distance cut-off and combined mutants with the highest stability; the lack of additivity here indicates possible epistatic interactions between residues in close proximity.

### Thermal aggregation analysis of stabilized mutants

**Figure 9:**
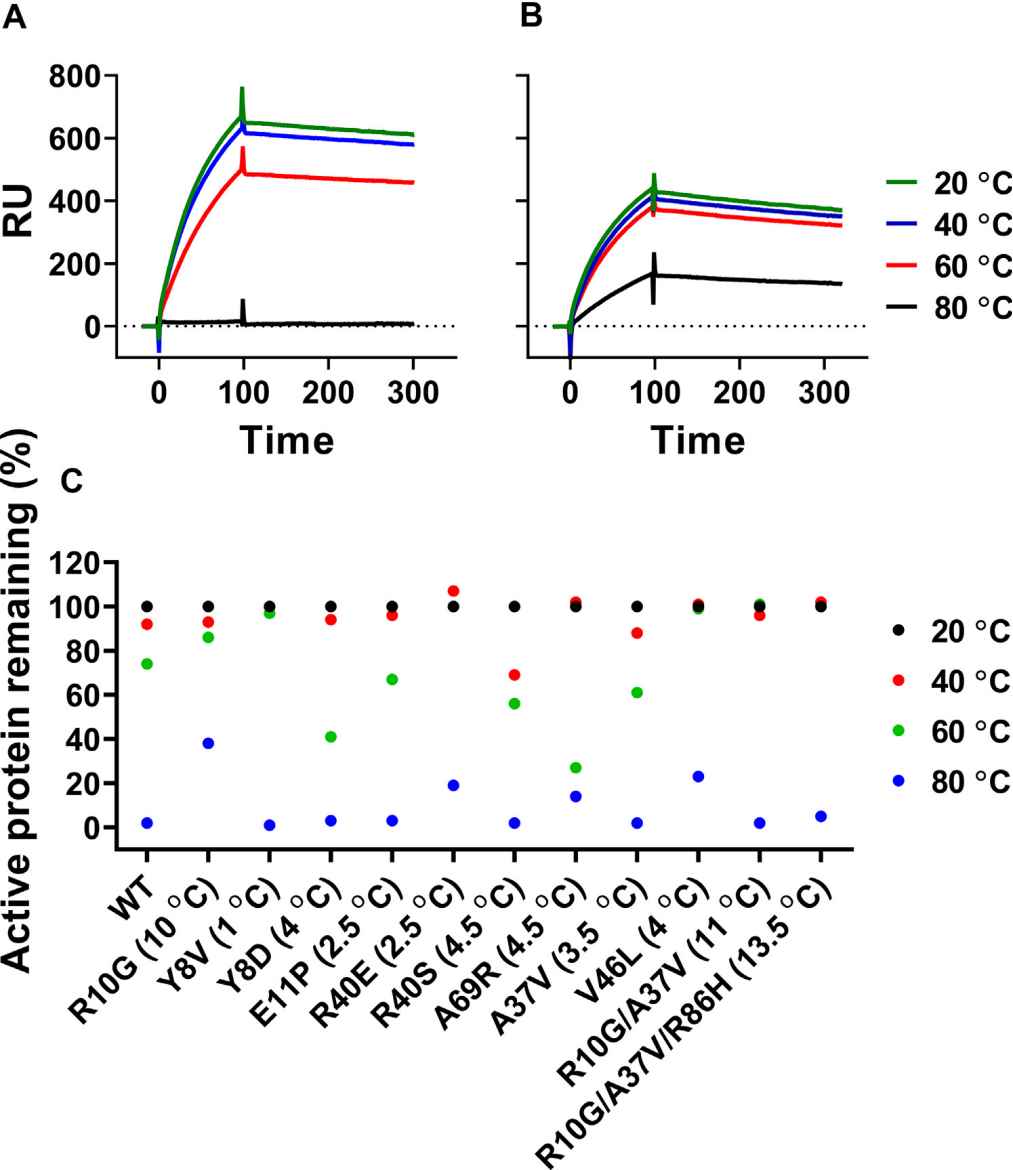
**Estimation of the fraction of active protein after thermal stress.** CcdB WT and mutants were incubated at 20, 40, 60, and 80 ^°^C for 1 h. The fraction of active protein was subsequently estimated by assaying binding to GyrA at 25 ^°^C using SPR. A representative SPR sensorgram for (*A*) WT CcdB and (*B*) R10G CcdB showing the relative amount of active protein remaining in the samples after incubation at different temperatures for 1 h. *C*, Fraction (%) of active protein after incubation at indicated temperature for 1 h. Δ*T*_m_ of stabilized mutants is mentioned next to the key of each mutant. SPR, surface plasmon resonance

To ascertain the ability of mutations to prevent loss of function from transient high temperature exposure, CcdB mutants were incubated at elevated temperatures for 1 h; this can result in unfolding of CcdB and subsequent irreversible aggregation (41). The fraction of active protein remaining after incubation was assessed by its ability to bind GyrA at room temperature using SPR. In the case of WT, we did not observe a large decrease in the fraction of the active protein except when incubated at temperatures at and above 60 °C (Fig. 9*A*). After incubation at 80 °C, the protein was completely denatured, and did not show any binding with gyrase. In contrast, one stabilized single mutant R10G and three multi-site mutants retained significant activity after incubation at 80 °C for 1 h (Fig. 9*B*). Six mutants showed a higher fraction of the active protein than WT after heating at 60 °C (Fig. 9*C*). Three single mutants and two double mutants showed no reduction in the active fraction of protein. Surprisingly, the triple mutant (R10G/A37V/R86H), which showed higher thermal stability than the R10G single mutant, showed higher aggregation at 80 °C, unlike R10G, which was partially resistant to aggregation under these conditions. This shows that thermal stability and thermal tolerance need not always be correlated, and stability-enhancing and aggregation-preventing mutants and mutant combinations can be different.

### Sorting of multiple libraries simultaneously

**Figure 10:**
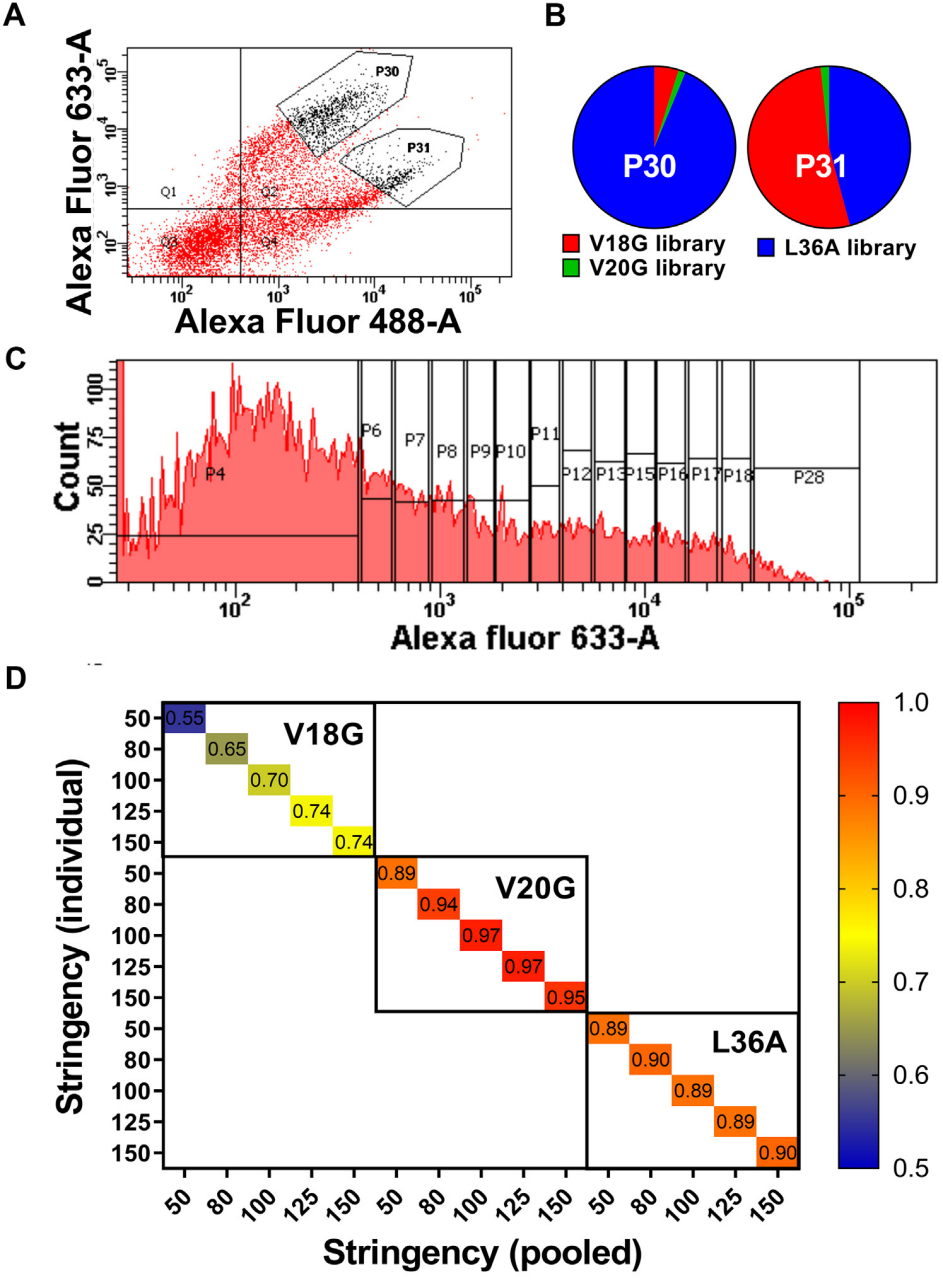
**FACS of pooled libraries.***A*, Dot plot showing the expression and binding of pooled library. Two different gates, P30 and P31, were used to sort the populations showing the highest expression and binding. *B*, Pie chart of relative enrichment of mutants from each library after one round of sorting and deep sequencing in gates P30 and P31. *C*, Sorting of pooled V18G, V20G, and L36A library based on binding to GyrA14. *D*, Heat map of correlation coefficient between the binding MFI of mutants calculated from an individual library and the pooled library at different stringencies, where stringency is the minimum number of reads per mutant. FACS, fluorescence-activated cell sorting.

When the suppressor mutations were identified using both enhanced binding to ligand relative to the corresponding PIM as well as alleviating the destabilizing effect of at least two PIMs as the criteria, predictions were highly specific. Hence, to find a larger number of stabilizing mutants, it is desirable to screen multiple PIM libraries. However, screening multiple individual libraries is laborious. In most FACS-based library screens, individual libraries are sorted for multiple rounds to enrich for the best binders. If this approach is applied to pooled libraries with multiple PIMs, the most stable PIM will dominate, resulting in the enrichment of mutants only from this library. To confirm this, we therefore sorted the pooled library as explained (Fig. 10*A*) and we found that mutants with the highest binding were largely from the L36A library as this PIM has the weakest effect on binding (Fig. 10*B*). To overcome this problem, we instead sorted multiple populations based on binding of the pooled library (Fig. 10*C*) and compared the relative binding of putative (PIM, suppressor) pairs with those of individual PIMs. We found a good correlation between the MFI_seq_ (bind) of mutants from the pooled library with those from individually analyzed libraries (Fig. 10*D*), with an increase in the correlation as the read cutoff increased. This suggests that single-round sorting of YSD-pooled SSSM libraries can rapidly identify stabilized mutations.

## Discussion

Directed evolution has drastically reduced the time required to design proteins with new activities. Directed evolution is performed most often in conjunction with display techniques such as phage and YSD, which involve selection of better binders to a target of interest from large libraries. While this approach readily selects for high-affinity binders, selecting for stable proteins is more difficult. Phage display libraries have very high diversity (42). However, in phage display experiments, there is less control over the selection of populations during enrichment and effects of post-translational modification including glycosylation cannot be studied. Yeast display enables the selection of populations during enrichment using FACS (5). In a recent report, expression level following YSD was used to identify a few stabilized mutants of SARS CoV-2 RBD (39). In the present study, we did not see a good correlation between the expression level of individual mutants and thermal stability of stabilized mutants. Instead, we observed that MFI_seq_ (bind) is a better predictor of protein stability than MFI_seq_ (expr). Previously, we found that for stable proteins it is difficult to isolate stabilizing mutants using YSD, as the surface expression and binding of all the mutants above threshold stability are often similar (22). To overcome this problem, we introduced a PIM in the DMS library of CcdB which reduced binding of all the mutants present in the library and then selected for suppressors which showed improved binding compared to the PIM. We hypothesized that if a stabilizing mutation alleviates the destabilizing effect of at least two PIMs, it is likely to be a true global suppressor. We therefore introduced four different PIMs with varied stability in the DMS library.

We also compared our stabilized mutant prediction with the *in silico* tools DeepDDG (34), PremPS (35), PoPMuSiC (36), INPS-MD (37) and PROSS (43). DeepDDG, PremPS, PoPMuSiC, and INPS-MD did not provide a good estimation of the stability of the stabilized mutants. PROSS is an alternative approach, which uses consensus sequence analysis combined with ROSETTA energy calculations, to predict stabilized protein sequences which contain a large number of (possibly) small effect mutations. PROSS necessarily requires a large number of sequences in a multiple sequence alignment. Also, for some applications, notably in vaccine immunogen design, it is desirable to achieve stabilization through a minimal number of substitutions so as to minimize unwanted changes in surface amino acids that might negatively impact immunogenicity, which is different from and complementary to the PROSS approach.

For libraries with the V18G, V20G, and L36A PIMs used in the study, a high specificity for the prediction was observed. These PIM libraries also showed a good correlation between protein stability and MFI_seq_ (bind) (Fig. 2). The identified mutants also showed additive stabilization when double or triple mutants were combined.

We also found that if a greater number of PIM libraries are screened, it enhances the prediction of stabilized mutants. To decrease the time and effort involved in screening multiple libraries, we pooled multiple libraries. The pooled library showed similar reconstructed MFIs of mutants to those obtained from individually analyzed libraries, validating the approach used. Importantly, the size of libraries used here is relatively small, identical to DMS libraries, with a total size of 32 N, where N is the length of the protein sequence. It can thus easily be extended to other library technologies, including lentiviral and transposon libraries, phage and mammalian display.

The methodology has the following limitations. The methodology requires a conformation-specific ligand to differentiate between the destabilized PIM and stabilized PIM–suppressor pair. For some proteins, hyperglycosylation on the yeast cell surface may interfere with the binding between YSD protein and its ligand. As an alternative to ligand binding, protease resistance can also be potentially used to select for stabilized mutants (44, 45). However, the aggregation and unfolding on the yeast cell surface may limit the cleavage of such proteins. Use of a protease assay to screen for stabilized mutants assumes that protease cleavage occurs primarily in the unfolded state (46). While this is true for small well-folded proteins, for larger proteins, initial sites of cleavage may occur at surface loops, complicating interpretation of protease screening results. Combing individual mutants from deep mutational scans has limitations, and there are often trade-offs between enhancements in stability and binding (47). However, by combining putative stabilizing mutations which are not close to each other or active-site residues, we believe that significant stabilization is possible without negatively impacting binding affinity as we have demonstrated both for CcdB in the present work and for the RBD for SARS-CoV-2 in another study (38). Overall, the present methodology offers a robust pathway to identify stabilizing mutations in any protein of interest for which a surface display based binding screen is available.

## Experimental procedures

### Bacterial strains, yeast strains, and plasmids

*E. coli* strain Top10Gyrase has a mutation in the gyrA gene which prevents CcdB toxicity. The EBY100 strain of *Saccharomyces cerevisiae* has TRP1 mutation which makes it auxotrophic for tryptophan, and transformants can be selected on minimal media. WT and mutant *ccdB* genes were coned in pBAD24 plasmid for controllable expression in *E.coli*. pPNLS shuttle vector was used to display CcdB mutants for yeast cell surface expression.

### Purification of wild-type and mutant CcdB proteins

CcdB WT and mutant proteins were purified as described (48); briefly, overnight grown culture was diluted 100 folds in 300 ml of LB media containing ampicillin (100 μg/ml). The cells were grown and induced at an A_600_ ∼0.5 for 3 h at 37 °C. The cells were harvested after induction and lysed using sonication in lysis buffer (10 mM Hepes, 1 mM EDTA, 10% glycerol, pH 8). The soluble fraction was separated using centrifugation and incubated with Affigel-15 coupled to CcdA peptide (residue 45–72) for 2 h at 4 °C. The unbound fraction was removed, and beads were washed with bicarbonate buffer (50 mM NaHCO_3_ and 500 mM NaCl). The proteins were eluted with glycine (200 mM, pH 2.5) and collected in tubes containing an equal volume of Hepes buffer (400 mM, pH 8).

### Single-site saturation suppressor mutagenesis library generation

A CcdB DMS library in which each individual residue was randomized, was generated through an inverse PCR-based approach (49). Briefly, for a given site, the forward primer had NNK (where K is G or T) at the 5′ end of the primer and the reverse primer starts at the -1 site, relative to the mutation. Individual single-site mutants were generated by inverse PCR, pooled in equimolar ratio, gel extracted, phosphorylated, and blunt end ligated at 15 °C. The ligated product was column purified and transformed in electrocompetent *E. coli* Top10Gyrase cells. Transformed colonies were scraped, and pooled plasmids were purified. The second site suppressor mutant library for CcdB was generated by introducing PIMs in the DMS library as described (29). Briefly, for a given PIM introduction, WT CcdB and CcdB library were amplified in two fragments using two sets of oligos. For each fragment, one of the oligos binds to the vector and the other binds to the gene. The primers of both fragments which bind to the gene were completely overlapping and contained the desired PIM mutation. Two separate transformations were performed in yeast (50); in the first transformation, fragment 1 of the CcdB library was combined with fragment 2 of WT CcdB, and in the second transformation, fragment 2 of the CcdB library was combined with fragment 1 of WT CcdB. The transformed cells were scraped and pooled based on the number of mutants present in each transformed plate in an attempt to ensure equal representation of all (PIM, mutant) pairs in the resulting library.

### Yeast surface expression and sorting of single-**site saturation suppressor mutagenesis** library

YSD and flow cytometric analysis were performed as explained earlier (22). Briefly, *S. cerevisiae* EBY100 cells containing WT CcdB or mutant plasmids were grown in SDCAA (glucose 20 g/L, yeast nitrogen base 6.7 g/L, casamino acid 5 g/L, citrate 4.3 g/L, and sodium citrate dihydrate 14.3 g/L) media for 16 h and induced in SGCAA (galactose 20 g/L, yeast nitrogen base 6.7 g/L, casamino acid 5 g/L, citrate 4.3 g/L, and sodium citrate dihydrate 14.3 g/L) media for an additional 16 h at 30 °C. Ten million cells were taken for FACS sample preparation. The SSSM library of CcdB was sorted based on 1D sorting of surface expression and binding. The cells were incubated with 200 μl of chicken anti hemagglutinin antibodies (Bethyl Laboratories, 1:600 dilution), followed by incubation with 200 μl of goat anti-chicken antibodies (Invitrogen, 1:300 dilution) conjugated with Alexa Fluor 488 to sort the cells based on the cell surface expression. The induced cells were incubated with 200 μl of FLAG-tagged GyrA14 (1000 nM). The estimated K_d_ for WT CcdB to GyrA14 is 4 nM. A higher CcdB concentration was employed to ensure that even destabilized mutants where only a small fraction was properly folded, showed detectable binding to CcdB. The cells were washed and incubated with 200 μl of mouse anti-FLAG antibodies (Sigma, 1:300 dilution), followed by incubation with 200 μl of rabbit anti-mouse antibodies (Invitrogen, 1:1500 dilution) conjugated with Alexa Fluor 633 to sort the cells based on binding of the displayed CcdB mutant to the cognate ligand, GyrA14 (29). The sorting of CcdB libraries was performed using a BD Aria III cell sorter. In the case of simultaneous sorting of multiple SSSM libraries, each library sample was prepared separately as explained previously and pooled before sorting.

### Sample preparation for deep sequencing

Deep sequencing samples were prepared as explained earlier (22). Briefly, sorted cells were grown on SDCAA agar plates, colonies were scraped, and pooled plasmids were extracted. The ccdB gene was PCR amplified using the primers having multiplex identifier (MID) sequence at the 5′ end, that bind upstream and downstream of the ccdB gene to segregate the reads from different sorted bins. The DNA was PCR amplified for 15 cycles; equal amounts of DNA from each sorted population were pooled, gel extracted, and the library was generated using TruSeq DNA PCR-Free kit from Illumina. The sequencing was done on an Illumina HiSeq 2500 250PE platform at Macrogen.

### Analysis of deep sequencing data

Deep sequencing data for the CcdB mutants were processed as described (22). Briefly, the paired end reads were assembled using the PEAR, version 0.9.6 (Paired-End Read Merger) tool (51). Following assembly, reads were filtered to eliminate those that do not contain the relevant MID and/or primers along with the reads having mismatched MIDs. Only those reads that have bases with a Phred score ≥20 are retained. Reads that pass the assembling and filtering step were binned according to the respective MIDs. Binned reads were aligned with the WT ccdB sequence using the Water, version 6.4.0.0, program (52) and reformatted. Finally, the reads were classified based on insertions, deletions, and substitutions (single, double, etc. mutants).

### Mean fluorescent intensity reconstruction from deep sequencing data

The MFI of each mutant was reconstructed as described (22). Briefly, reads of each mutant were normalized across different bins (Equation 1). The fraction of each mutant (X*i*) distributed across each bin was calculated (Equation 2). The reconstructed MFI (MFI_seq_) of individual mutant was calculated by the summation of the product, obtained upon multiplying the fraction (X*i*) of that mutant in bin (*i*) with the MFI of the corresponding bin obtained from the FACS experiment (F*i*), across the various bins populated by that mutant (Equation 3). The normalized MFI of each mutant was calculated from the reconstructed MFI of each mutant (Equation 4).

(1) $$\text{Normalized read of mutant in bin }i\;\left(\text{N}i\right)\;=\;\frac{No.\;of\;mutant\;i\;in\;bin\;i}{\sum Reads\;in\;bin\;i}$$

(2) $$\text{Fraction of mutants in each gate }\left(\text{X}i\right)\;=\;\frac{Ni}{\sum_{1}^{n}Ni}$$

(3) $$\text{Reconstructed MFI }=\sum_{1}^{n}Fi*Xi$$

(4) $$\text{Normalized MFI }=\;\frac{Reconstructed\;MFI\;of\;mutant\;i}{Reconstructed\;MFI\;of\;WT}$$

MFI_seq_ (expr) and MFI_seq_ (bind) refer to reconstructed values from FACS sorting based on mutant expression and binding to GyrA14, respectively. Stabilized mutants were classified as those that showed at least 25% enhanced binding or expression when present as the PIM–suppressor pair compared to the PIM alone.

### Protein thermal stability measurement

This was carried out as described (48). Briefly, a solution of total volume 20 μl containing 10 μM of the purified CcdB protein and 2.5× SYPRO Orange dye in buffer (200 mM Hepes, 100 mM glycine), pH 7.5, was heated from 15 °C to 90 °C with 0.5 °C increment every 30 s on an iCycler iQ5 Real Time Detection System (Bio-Rad). The normalized fluorescence data were plotted against temperature (53).

### Thermal aggregation studies of CcdB mutants

CcdB mutants and WT proteins (500 nM, 200 μl for each of the proteins) were subjected to incubation at four different temperatures, 20, 40, 60, and 80 °C, on an iCycler iQ5 Real Time Detection System (Bio-Rad). The temperature was gradually increased to the desired temperature at a rate of 3 °C/min, and samples were kept at the desired temperature for 1 h. The heated protein was then cooled down to 4 °C at the rate of 3 °C/min. The aggregated protein was removed using centrifugation at 18000*g*. The fraction of active protein remaining was measured by binding to GyrA14 on a Biacore 2000 SPR platform. The percentage of active protein at different temperatures was calculated using the following equation:

$$\%Active=\;\frac{Binding\;of\;CcdB\;after\;incubation\;at\;temperature\;T\;\left(RU\right)}{Binding\;of\;CcdB\;after\;incubation\;at\;20{}^{\circ}\text{C }\;\left(RU\right)}*100$$

### Statistical analysis

All the data were plotted using the GraphPad Prism software, version 9.0.0. The correlation coefficients between deep sequencing replicates were estimated using the GraphPad Prism software, version 9.0.

## Data availability

The deep sequencing data discussed in the present study have been deposited in NCBI’s Sequence Read Archive (accession no. SRR16094780). Illumina sequencing counts for each ccdB double mutant of FACS bins are available at https://github.com/rvaradarajanlab/ccdb_sssm/blob/main/ccdn_sssm_freq.xlsx. MFI_seq_ (expr) and MFI_seq_ (bind) of CcdB mutants are available at https://github.com/rvaradarajanlab/ccdb_sssm/blob/main/Supplementary_data_sssm_calc_MFI.xlsx. Remaining data are available in the manuscript.

## Supporting information

This article contains supporting information.

## Conflict of interest

The authors claim no conflict of interest.
