# Supplementary material for: Identification of stabilizing point mutations through mutagenesis of destabilized protein libraries
Source: J Biol Chem. 2022 Mar 3;298(4):101785. doi: 10.1016/j.jbc.2022.101785 (PMC8971944; doi:10.1016/j.jbc.2022.101785)
Supplement: Supporting information Figures S1–S3 and Tables S1–S3 [file mmc1.docx]

**Identification of stabilizing point mutations through mutagenesis of destabilized protein libraries.**

**Shahbaz Ahmed^1^, Kavyashree Manjunath^2^, Gopinath Chattopadhyay^1^, Raghavan Varadarajan^1,*^**

^1^Molecular Biophysics Unit, Indian Institute of Science, Bangalore-560012, India

^2^ Institute of Stem Cell Science and Regenerative Medicine, Bangalore 560065, India

*Author for correspondence

Telephone: +91-80-2293-. 2612

Fax: +91-80-23600535

**Email**: [varadar@iisc.ac.in](mailto:varadar@iisc.ac.in)

**This file contains**

**Supporting Figure S1-S3**

**Supporting Table S1-S3**

**Supporting Information**


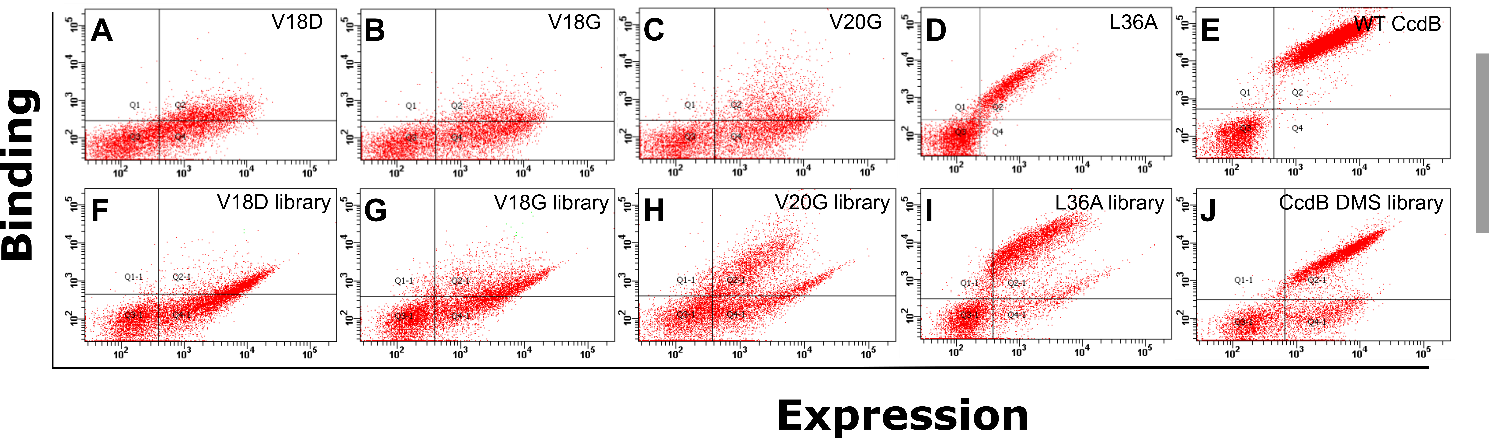


**Figure S1: FACS double plot showing the expression and binding to GyrA14 of CcdB PIMs, WT and their corresponding DMS libraries.** FACS double plot of CcdB PIMs (A) V18D, (B) V18G, (C) V20G and (D) L36A and (E) WT CcdB. Double plots of DMS libraries in the background of (F) V18D, (G) V18G, (H) V20G (I) L36A (J) WT CcdB


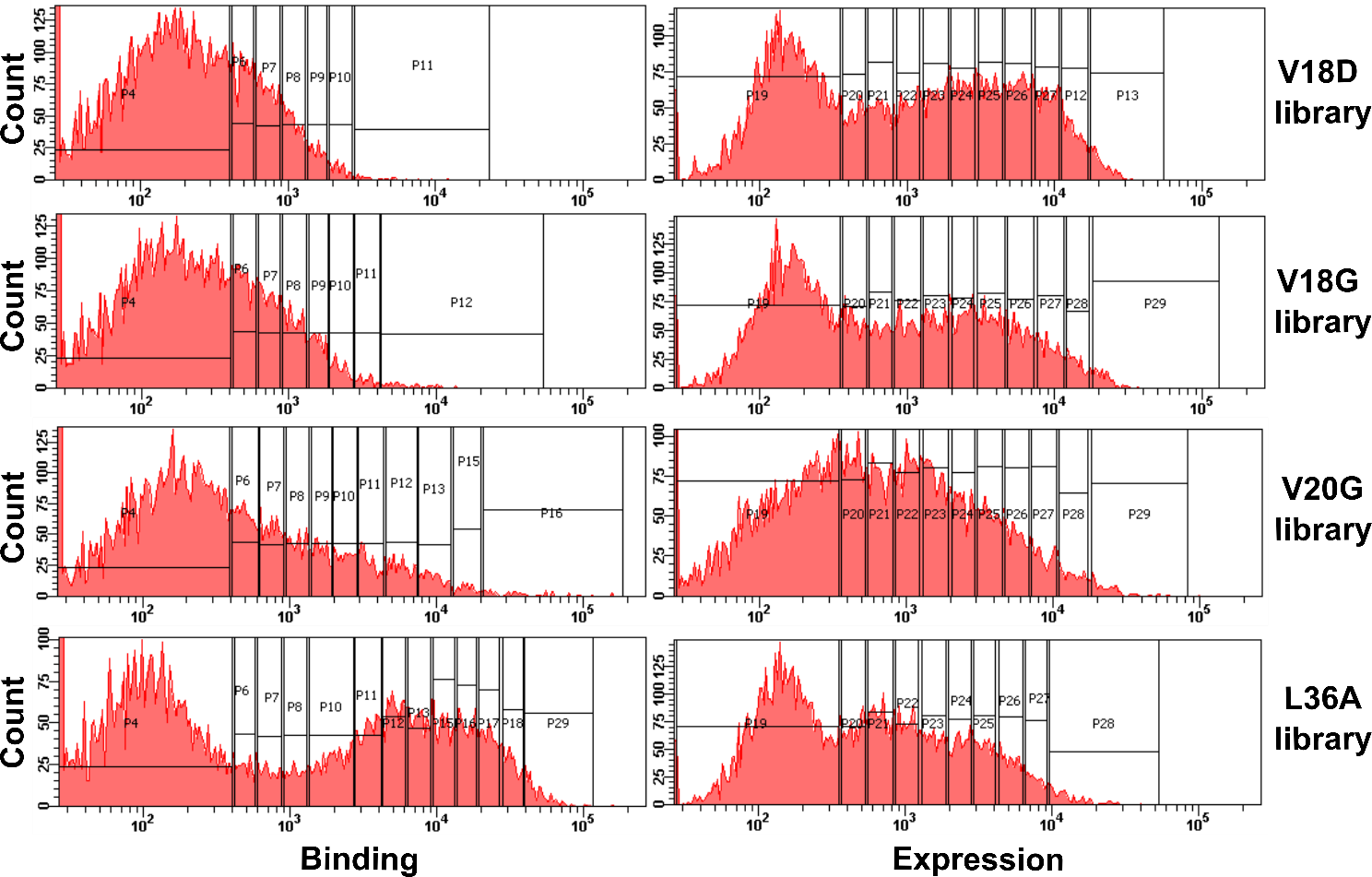


**Figure S2: Histograms of expression and binding to GyrA14 of PIM containing CcdB libraries.** Left and right panels show binding and expression histograms. Different populations sorted into bins based on expression and binding are indicated by the vertical lines.


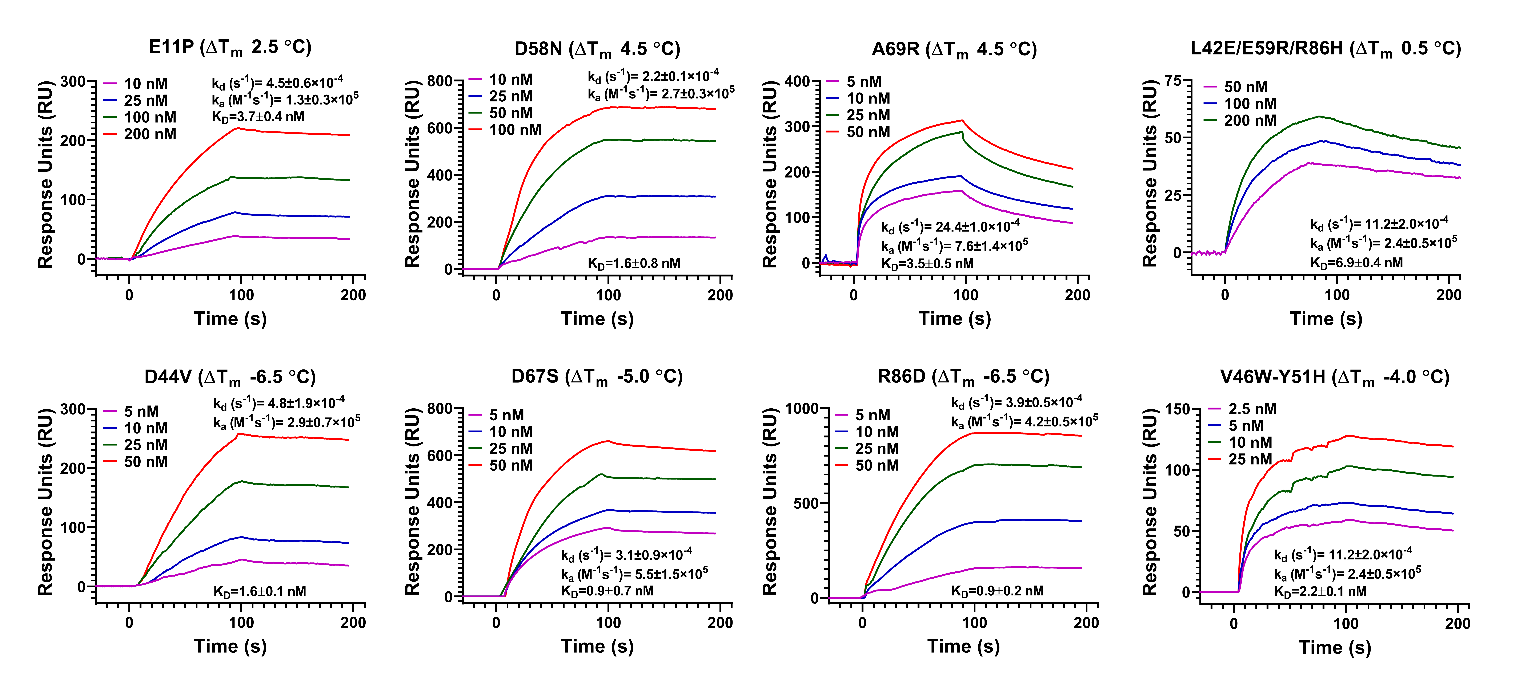


**Figure S3: Binding of GyraseA-14 to CcdB mutant proteins**. The ligand GyrA14 was immobilized on the CM5 chip by standard amine coupling. Binding was measured by passing varying concentrations of the analyte (CcdB proteins) over the ligand (GyrA14) immobilised chip. Overlays show the binding kinetics of ligand with different concentrations of analyte. Top panels shows the binding kinetics of stabilized single or multi-mutants, bottom panel shows the binding kinetics of destabilized single or multi-mutants. The data was fitted to the 1:1 Langmuir interaction model to obtain the kinetic parameters.

.

**Table S1: Number of mutants analysed in each PIM library for expression and binding.**

|  |  | **Binding** | **Expression** |
| --- | --- | --- | --- |
| **V18D library** | **Codon level** | **1275** | **1139** |
|  | **Codon averaged** | **935** | **862** |
| **V18G library** | **Codon level** | **1319** | **1353** |
|  | **Codon averaged** | **967** | **989** |
| **V20G library** | **Codon level** | **1237** | **1343** |
|  | **Codon averaged** | **890** | **977** |
| **L36A library** | **Codon level** | **1763** | **670** |
|  | **Codon averaged** | **1189** | **530** |

**Table S2: Thermal stability of CcdB mutants estimated using TSA.**

| Mutant | ΔT_m_ | Mutant | ΔT_m_ | Mutant | ΔT_m_ | Mutant | ΔT_m_ |
| --- | --- | --- | --- | --- | --- | --- | --- |
| R62P | -24 | D67S | -5 | P28N | -1.5 | Y8L | 2 |
| I56P | -17 | I25A | -4.5 | M32V | -1.5 | R10V | 2 |
| P35W | -15.5 | I25N | -4.5 | V46G | -1.5 | I24L | 2 |
| L50N | -15 | S43Y | -4 | G57C | -1.5 | I24Y | 2 |
| V54S | -15 | V46R | -4 | E59Q | -1.5 | V33M | 2 |
| P52S | -14.5 | S47R | -4 | A69V | -1.5 | E11P | 2.5 |
| L50R | -14 | V53W | -4 | V75G | -1.5 | R40E | 2.5 |
| T7P | -13 | I25T | -3.5 | S12A | -0.5 | L42E | 2.5 |
| D82I | -13 | S38K | -3.5 | A37D | -0.5 | S60M | 2.5 |
| D67T | -11.5 | D44H | -3.5 | A37Y | -0.5 | L42V | 3 |
| L36V | -10 | R86E | -3.5 | S43A | -0.5 | R48T | 3 |
| T65Q | -9.5 | Y14A | -3 | WT | 0 | 101N | 3 |
| D44F | -7 | L42K | -3 | Y14W | 0 | A37V | 3.5 |
| M64P | -7 | L50I | -3 | S47M | 0 | H55R | 3.5 |
| G77P | -7 | D67L | -3 | K9R | 0.5 | S84G | 3.5 |
| D44V | -6.5 | K9M | -2.5 | K45Y | 0.5 | Y8D | 4 |
| P72L | -6.5 | D26S | -2.5 | V46W | 0.5 | I24K | 4 |
| R86D | -6.5 | R30V | -2.5 | R48G | 0.5 | V46L | 4 |
| A39R | -6 | S38G | -2.5 | A69T | 0.5 | R86H | 4 |
| E59P | -6 | L42W | -2.5 | R86K | 0.5 | S38N | 4.5 |
| V71A | -6 | R62G | -2.5 | Y8V | 1 | R40S | 4.5 |
| G77S | -6 | H55C | -2 | R13V | 1 | D58N | 4.5 |
| P28S | -5.5 | E59R | -2 | L41M | 1 | A69R | 4.5 |
| V53P | -5.5 | S60G | -2 | S43T | 1 | L41E | 5 |
| E59Y | -5.5 | S70Q | -2 | R10K | 1.5 | S60E | 7.5 |
| E79I | -5.5 | K9C | -1.5 | I24E | 1.5 | R10G | 10 |
| D26A | -5 | R13F | -1.5 | A37L | 1.5 |  |  |
| P28Y | -5 | D26V | -1.5 | V46Y | 1.5 |  |  |

**Table S3: PROSS predictions of CcdB stabilizing mutations.** For three designs PROSSS did not predict any mutation, for the remaining designs 2-16 mutations combinations were predicted. The mutations predicted by PROSS are highlighted with different colours where, grey colour indicates the mutants for which data is not available by saturation suppressor mutagenesis. Orange colour indicate active-site mutants, green colour indicates predicted stabilizing mutation both by saturation suppressor mutagenesis and PROSS, red colour indicates destabilizing mutation predicted by saturation suppressor mutagenesis and stabilizing mutation predicted by PROSS.

| Residue | Wild Type | Design 1 | Design 2 | Design 3 | Design 4 | Design 5 | Design 6 | Design 7 | Design 8 | Design 9 |
| --- | --- | --- | --- | --- | --- | --- | --- | --- | --- | --- |
| 49 | E |  |  |  |  |  |  |  |  | R |
| 24 | I |  |  |  |  |  |  |  |  | L |
| 92 | N |  |  |  |  |  |  |  |  | A |
| 13 | R |  |  |  |  |  |  |  |  | A |
| 47 | S |  |  |  |  |  |  |  |  | P |
| 58 | D |  |  |  |  |  |  |  | G | G |
| 87 | E |  |  |  |  |  |  |  | R | R |
| 57 | G |  |  |  |  |  |  |  | N | N |
| 15 | R |  |  |  |  |  |  |  | K | K |
| 37 | A |  |  |  |  |  |  | V | I | F |
| 88 | N |  |  |  |  |  |  | D | D | D |
| 95 | N |  |  |  |  |  |  | D | D | D |
| 41 | L |  |  |  |  | H | H | H | H | H |
| 60 | S |  |  |  |  | D | D | D | D | D |
| 38 | S |  |  |  | P | P | P | P | P | P |
| 61 | W |  |  |  | Y | Y | Y | Y | Y | Y |
